# The Selective Agonist for Sphingosine-1-Phosphate Receptors Siponimod Increases the Expression Level of *NR4A* Genes in Microglia Cell Line

**DOI:** 10.3390/cimb44030083

**Published:** 2022-03-07

**Authors:** Francesca Montarolo, Serena Martire, Fabiana Marnetto, Paola Valentino, Sabdi Valverde, Marco Alfonso Capobianco, Antonio Bertolotto

**Affiliations:** 1 Neuroscience Institute Cavalieri Ottolenghi (NICO), 10043 Orbassano, Italy; francesca.montarolo@unito.it (F.M.); serena.martire@gmail.com (S.M.); fabiana.marnetto@gmail.com (F.M.); paolaval81@hotmail.com (P.V.); valverdesabdi@gmail.com (S.V.); mcapobianco1972@gmail.com (M.A.C.); 2 Neurology Department and Regional Referring Center of Multiple Sclerosis (CReSM), University Hospital San Luigi Gonzaga, 10043 Orbassano, Italy; 3 Department of Molecular Biotechnology and Health Sciences, University of Turin, 10126 Turin, Italy; 4 Department of Neuroscience “Rita Levi Montalcini”, University of Turin, 10126 Turin, Italy

**Keywords:** multiple sclerosis, *NR4A*, NURR1, sphingosine-1-phosphate (S1P) receptors, siponimod, fingolimod, CNS resident cells

## Abstract

Fingolimod (FTY720) and siponimod (BAF312) are selective agonists for sphingosine-1-phosphate (S1P) receptors approved for the treatment of relapsing–remitting (RR) and secondary progressive (SP) multiple sclerosis (MS), respectively. BAF312 exerts pro-myelination and neuro-protective functions on CNS resident cells, although the underlying molecular mechanism is not yet fully understood. *NR4A*2 is an anti-inflammatory gene, belonging to the *NR4A* family, whose expression is reduced in blood from treatment-naïve patients with RRMS, but is restored in patients treated with FTY720 for more than two years. We performed an in vitro study to investigate the potential involvement of the *NR4A* genes in the protective and restorative effects of BAF312. We showed that BAF312 enhances the expression of *NR4A*1 and *NR4A*2 in the N9 microglial cell line, but has no effect in the peripheral blood mononuclear cells and oligodendrocytes. This study suggests a novel molecular mechanism of action for the selective agonists for S1P receptors within the CNS.

## 1. Introduction

Multiple sclerosis (MS) is a chronic autoimmune disease of the central nervous system (CNS) characterized by demyelination and subsequent axonal damage [1], in which inflammation, lymphocyte/macrophage infiltration, and microglia activation play a key pathophysiological role [1]. Among a variety of disease-modifying therapies (DMTs) approved for the treatment of MS, the selective agonists for sphingosine-1-phosphate (S1P) receptors, namely fingolimod (FTY720) and siponimod (BAF312), are a class of effective oral drugs [2]. S1P is a naturally occurring lipid mediator binding five cell-surface membrane G protein-coupled receptor subtypes named S1P1, S1P2, S1P3, S1P4, and S1P5. S1P1, S1P2, and S1P3 show broad tissue expression, while S1P4 and S1P5 are primarily expressed in the immune system and the CNS, mainly by lymphocytes and oligodendrocytes [3]. FTY720 was the first S1P receptor modulator approved for the treatment of relapsing–remitting (RR) MS [4,5], and it was found to activate all S1P receptor subtypes, except for S1P2. BAF312 is a selective agonist for S1P1 and S1P5 [2]; in the USA, it was approved for the treatment of relapsing forms of MS, including clinically isolated syndrome, RRMS, and secondary progressive (SP) MS, and in Europe for SPMS with clinical/radiological evidence of disease activity.

The immune-modulatory properties of selective agonists for S1P receptors involve the retention of T and B lymphocytes in secondary lymphoid organs through the internalization of the S1P1 present on their surface, thus preventing their recirculation to the CNS [6]. Numerous preclinical observations suggest that BAF312 has also pro-myelination and neuro-protective properties attributable to S1P1- and S1P5-dependent effects on CNS resident cells [7,8,9], although the underlying molecular mechanism is not yet fully understood.

We recently observed that patients with RRMS treated with FTY720 express higher levels of the nuclear receptor-related 1 protein (NURR1, also called *NR4A*2) in blood than untreated patients [10], while such a difference was not associated with treatment with other DMTs, such as interferon beta, glatiramer acetate, and natalizumab [11].

*NR4A*2 is a transcription factor belonging to steroid nuclear hormone receptor family 4, group A (*NR4A*), which also includes *NR4A*1 (neuron-derived clone 77; Nur77) [12] and *NR4A*3 (neuron-derived orphan receptor 1; Nor-1) [13]. *NR4A* is known to exert anti-inflammatory and neuro-protective functions through the inhibition of the pro-inflammatory transcription factor NF-kB, both in blood cells and in microglia [14]. Decreased *NR4A* transcript levels have been reported in blood from patients with RRMS compared to healthy controls (HC) [10,11,15,16,17,18,19], and a negative correlation between *NR4A*2 expression and the relapse rate and Expanded Disability Status Scale (EDSS) score has been described [11]. Furthermore, *NR4A*2 deficiency has been shown to revert in women with MS during pregnancy, which represents a state of immune tolerance associated with reduced disease activity [19]. Studies in murine models of MS also suggest a role for *NR4A*2 in the pathogenesis of the disease. In mice with Experimental Autoimmune Encephalomyelitis (EAE), *NR4A*2 deficiency advances the disease’s onset [20]. On the other hand, treatment with isoxazolo-pyridinone-7e, a potent activator of the *NR4A*2 signaling pathway, delays the EAE onset along with attenuating inflammation and neuro-degeneration in the spinal cord through an NF–kB pathway-dependent process [21]. Finally, Shaked and co-workers observed a worsening in the EAE condition after the *NR4A*1 germinal or conditional deletion in the myeloid cell population [22]. However, despite the accumulating evidence of a deregulation of *NR4A* in MS, its specific role in the pathogenesis of the disease is not yet fully understood.

In this study, we used cell mono-cultures to explore the ability of BAF312 treatment to modulate the expression of *NR4A* in blood cells, as already reported for FTY720 [10], and in CNS resident cells involved in the MS pathogenesis, such as microglia and oligodendrocytes.

## 2. Results 

### 2.1. BAF312 Does Not Influence the Expression of *NR4A* in Primary Cultures of Human Peripheral Blood Mononuclear Cells (PBMCs)

**Figure 1:**
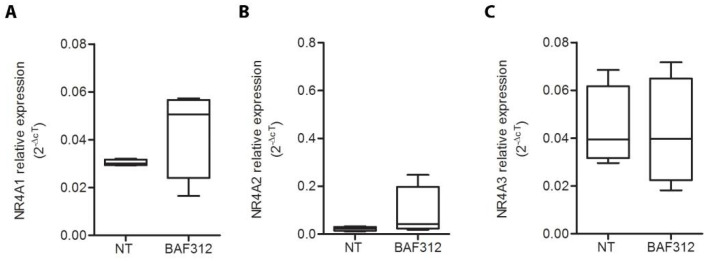
BAF312 does not influence the expression of the *NR4A* genes in primary cultures of PBMCs. Gene expression levels of *NR4A*1 (**A**), *NR4A*2 (**B**), and *NR4A*3 (**C**) in human primary cultures of PBMCs. Comparison of gene expression levels between not-treated (*n* = 4) and BAF312-treated (*n* = 4) cells. Two-tailed *t*-test for (**A**,**C**), and Mann–Whitney U test for (**B**).

Based on our previous results regarding FTY720 [10], we treated primary cultures of PBMCs extracted from human buffy coats with BAF312 to explore its effect on *NR4A* gene expression. Here, we observed that 24 h of in vitro stimulation with BAF312 is not able to modulate the expression level of *NR4A* (Figure 1A, *t*-test *p* = 0.249; Figure 1B, *t*-test *p* = 0.200; Figure 1C, *t*-test *p* = 0.898).

### 2.2. BAF312 Up-Regulates *NR4A*1, *NR4A*2, and TREM-2 Expression in N9 Microglial Cell Line

**Figure 2:**
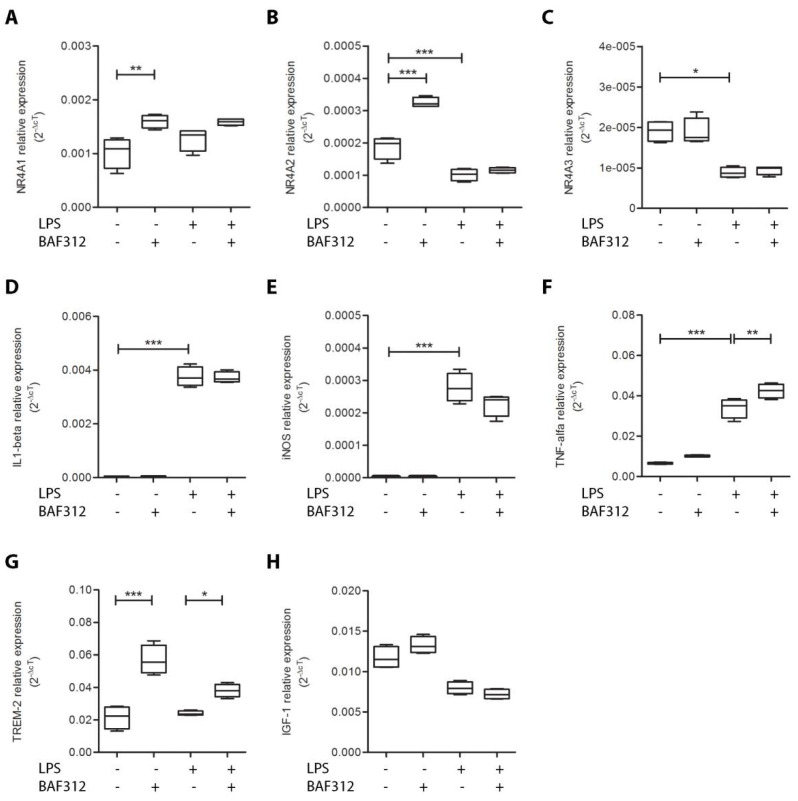
BAF312 up-regulates *NR4A*1, *NR4A*2, and TREM-2 expression in the N9 microglial cell line. Gene expression levels of *NR4A*1 (**A**), *NR4A*2 (**B**), *NR4A*3 (**C**), IL 1-beta (**D**), iNOS (**E**), TNF-alfa (**F**), TREM-2 (**G**), and IGF-1 (**H**) in N9 murine microglial cell line. Comparison of gene expression levels between not-treated (*n* = 4), BAF312 (*n* = 4), LPS (*n* = 4), and BAF312/LPS (*n* = 4) -stimulated cells. One-way ANOVA followed by Tukey’s HSD post hoc test for (**A**,**B**,**D**–**H**), and Kruskal-Wallis followed by Dunn’s post hoc test for (**C**). * 0.05 > *p* value ≥ 0.01; ** 0.01 > *p* value ≥ 0.001; *** *p* value < 0.001.

To evaluate whether the pro-myelination and neuro-protective effects of BAF312 could involve the modulation of the *NR4A* genes in microglia, we treated the N9 cell line with BAF312 in the presence or absence of LPS in order to mimic physiological and inflammatory conditions. In line with previous studies [23], we observed that stimulation with LPS decreased the expression levels of *NR4A*2 (Figure 2B, One-way ANOVA *p* < 0.0001, Tukey’s post hoc test *p* = 0.0005 for LPS stimulated vs. not treated) and *NR4A*3 (Figure 2C, Kruskal–Wallis test *p* = 0.01, Dunn’s post hoc test *p* = 0.0347 for LPS stimulated vs. not treated). We showed that BAF312 treatment increased the expression levels of *NR4A*1 under physiological conditions and, to a lesser extent, under inflammatory conditions (Figure 2A, One-way ANOVA *p* = 0.003, Tukey’s post hoc test *p* = 0.006 for BAF312 treated vs. not treated; Tukey’s post hoc test *p* = 0.139 for BAF312/LPS stimulated vs. LPS stimulated). BAF312 treatment strongly increased the expression levels of the *NR4A*2 gene only under physiological conditions (Figure 2B, One-way ANOVA *p* < 0.0001, Tukey’s post hoc test *p* < 0.0001 for BAF312 treated vs. not treated; Tukey’s post hoc test *p* = 0.796 for BAF312/LPS stimulated vs. LPS stimulated), while it had no effect on *NR4A*3 (Figure 2C, Kruskal–Wallis test *p* = 0.01, Dunn’s post hoc test *p* = 1.000 for BAF312 treated vs. not treated; Dunn’s post hoc test *p* = 0.919 for BAF312/LPS stimulated vs. LPS stimulated).

To characterize the molecular phenotype associated with BAF312 treatment, we also evaluated the gene expression levels of three pro-inflammatory microglial molecules, i.e., interleukin1-β (IL1-beta), inducible nitric oxide synthase (iNOS), and tumor necrosis factor-α (TNF-alfa). We first observed that the expression levels of these molecules increased after the stimulation with LPS, indicating the achievement of an inflammatory state (Figure 2D, One-way ANOVA *p* < 0.0001, Tukey’s post hoc test *p* < 0.0001 for LPS stimulated vs. not treated; Figure 2E, One-way ANOVA *p* < 0.0001, Tukey’s post hoc test *p* < 0.0001 for LPS stimulated vs. not treated; Figure 2F, One-way ANOVA *p* < 0.0001, Tukey’s post hoc test *p* < 0.0001 for LPS stimulated vs. not treated). BAF312 treatment showed no effect on IL1-beta and iNOS under both physiological and inflammatory conditions (Figure 2D, One-way ANOVA *p* < 0.0001, Tukey’s post hoc test *p* = 0.999 for BAF312 treated vs. not treated; Tukey’s post hoc test *p* = 0.997 BAF312/LPS stimulated vs. LPS stimulated; Figure 2E, One-way ANOVA *p* < 0.0001, Tukey’s post hoc test *p* = 1.000 for BAF312 treated vs. not treated; Tukey’s post hoc test *p* = 0.095 BAF312/LPS stimulated vs. LPS stimulated). On the other hand, treatment with BAF312 increased the expression levels of TNF-alfa only in the LPS-stimulated cells (Figure 2F, One-way ANOVA *p* < 0.0001, Tukey’s post hoc test *p* = 0.381 for BAF312 treated vs. not treated; Tukey’s post hoc test *p* = 0.008 for BAF312/LPS stimulated vs. LPS stimulated).

Finally, we investigated the effect of BAF312 treatment on the expression levels of the triggering receptor expressed on myeloid cells 2 (TREM-2), which plays a role in the CNS homeostasis [24,25], and of insulin growth factor 1 (IGF-1), known to be a neuro-protective microglial molecule [26]. BAF312 induced TREM-2 gene expression in both resting and LPS-activated microglia (Figure 2G, One-way ANOVA *p* < 0.0001, Tukey’s post hoc test *p* < 0.0001 for BAF312 treated vs. not treated; *p* = 0.0278 for BAF312/LPS stimulated vs. LPS stimulated), while it had no effect on IGF-1 expression (Figure 2H, One-way ANOVA *p* < 0.0001, Tukey’s post hoc test *p* = 0.175 for BAF312 treated vs. not treated; Tukey’s post hoc test *p* = 0.694 for BAF312/LPS stimulated vs. LPS stimulated).

### 2.3. BAF312 Does Not Influence the Expression of *NR4A* in Oligodendrocytic Cell Line

**Figure 3:**
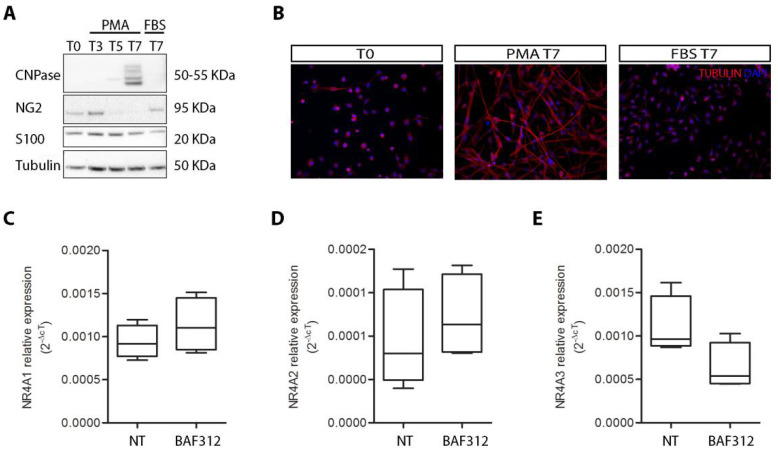
BAF312 does not influence the expression of the *NR4A* genes in the oligodendrocytic cell line. (**A**) Representative Western blots of CNPase, NG2, and S100 proteins extracted from the MO3.13 oligodendrocytic cell line under control conditions (T0), after differentiation with PMA for 3, 5, and 7 days in vitro (T3, T5, T7 PMA), and with FBS for 7 days in vitro (FBS T7). Tubulin served as the loading control. (**B**) Representative images of the MO3.13 oligodendrocytic cell line under control conditions (T0), after differentiation with PMA (T7 PMA) and FBS (FBS T7) for 7 days in vitro, and stained with tubulin antibody (red). DAPI (blue) counterstained the cell nuclei. Magnification 20×. Gene expression levels of *NR4A*1 (**C**), *NR4A*2 (**D**), and *NR4A*3 (**E**) in the MO3.13 oligodendrocytic cell under the PMA T7 condition. Comparison of the gene expression levels between the not-treated (*n* = 4) and BAF312-treated (*n* = 4) cells. Two-tailed *t*-test.

To evaluate whether the pro-myelination and neuro-protective effects of BAF312 could involve the modulation of *NR4A* in oligodendrocytes, we differentiated MO3.13 oligodendrocyte precursors into oligodendrocytes and treated them with BAF312. We first evaluated the cell differentiation state by Western blot (Figure 3A) and immunofluorescence (Figure 3B) analysis. After 7 days of culture in differentiation medium (PMA T7), the levels of the oligodendrocyte marker 20,30-cyclicnucleotide-30-phosphodiesterase (CNPase) increased (Figure 3A), while the expression of the astrocyte marker calcium-binding protein S100, as well as the oligodendrocyte precursor cell marker neural/glial antigen 2 (NG2), decreased (Figure 3A), supporting the achievement of a more mature oligodendrocyte state. On the other hand, when MO3.13 progenitor cells were cultured for 7 days in the presence of 10% FBS, but in the absence of PMA (FBS T7), S100 and NG2 were still detectable, while CNPase was undetectable (Figure 3A). The differentiation state of oligodendrocytes was also supported by the achievement of a branched morphology, which only characterized MO3.13 cells cultured in the differentiation medium (PMA T7) (Figure 3B). Finally, we analyzed the expression levels of the *NR4A* genes in PMA T7 MO3.13 oligodendrocytes, and we observed for the first time that they were expressed in the mature oligodendrocyte cell line. However, treatment with BAF312 did not modulate the expression levels of the *NR4A* genes (Figure 3C *t*-test *p* = 0.337, Figure 3D *t*-test *p* = 0.490 and Figure 3E *t*-test *p* = 0.081).

## 3. Discussion

FTY720 and BAF312 are selective agonists for S1P receptors, a class of promising DMTs currently in use for the treatment of MS [2]. Their effects are mediated through one or more of the five S1P receptor subtypes, which are expressed differently on different cell types, including lymphocytes, microglia, and oligodendrocytes [3]. FTY720 and BAF312 are characterized by different affinities for the S1P receptor subtypes, and while FTY720 was the first effective S1P receptor-modulating drug to be used in patients with RRMS, BAF312 was also proven to be clinically effective in patients with active SPMS [2]. Besides the known immune-modulatory effects exerted through the induction of S1P1 internalization in lymphocytes, BAF312 has also shown pro-myelination and neuro-protective effects due to the interaction with S1P1 and S1P5 on CNS resident cells [7,8,9]. However, the cell-specific molecular mechanisms responsible for this competence within the CNS are not yet completely understood.

*NR4A* is a family of steroid nuclear hormone receptors with anti-inflammatory and neuro-protective roles [14], and increasing evidence suggests their implication in the pathogenesis of MS [10,11,15,17,19]. We showed that treatment-naïve patients with RRMS have lower blood transcript levels of *NR4A*2 compared to HC [11,18,19], while this deficit is no longer evident in patients treated with FTY720, whose *NR4A*2 levels are similar to those of HC [10]. The recent approval of BAF312 for the treatment of active SPMS has prompted our interest in exploring for the first time its potential modulatory effect on the expression levels of the *NR4A* genes in both blood and CNS resident cells.

First, we investigated whether BAF312 was able to modify the expression of the *NR4A* genes in peripheral blood cells, as one might suppose based on our previous study in patients with MS treated with FTY720 [10]. We showed that 24 h of in vitro treatment with BAF312 did not modulate the *NR4A* gene expression in human PBMCs. However, this does not exclude the possibility that modulation could be observed after a longer in-vivo treatment, as, in our previous study, we examined patients treated with FTY712 for more than two years [10].

We then explored the effect of BAF312 on the gene expression levels of CNS resident cells that play a key pathogenic role in MS, namely microglia and oligodendrocytes. Microglia play a crucial role, not only in RRMS, but also in the progressive form of the disease, which is the particular target of BAF312. An elevated number of activated microglial cells has been found in the CNS of MS patients, in the active lesions that are characteristic of the RR form, as well as in the slowly expanding chronic active lesions that are characteristic of the progressive form of MS [27]. Notably, recent evidence suggests that, in addition to anti-inflammatory functions in blood, *NR4A*2 also exerts neuro-protective functions acting directly in microglia [28,29,30]. In fact, it has been demonstrated that *NR4A*2 expressed in microglia interacts with the NF-κB pathway, reducing the production of inflammatory and neurotoxic mediators and resulting in protective effects on neurons [14,31]. Here, we observed that the in vitro treatment of the N9 microglial cell line with BAF312 increased the expression level of the *NR4A*1, *NR4A*2, TREM-2, and TNF-alfa genes, suggesting that they may be involved in the beneficial effects of BAF312 in CNS resident cells. In particular, it has been suggested that increased levels of TREM-2, which is primarily expressed in the brain by microglia [32], facilitates recovery from brain injury [24,25]. In a murine model of CNS demyelination, antibody-dependent TREM-2 activation in microglia promoted the clearance of myelin debris, thus enhancing remyelination and axonal integrity [33]. The finding of increased expression of the TNF-alfa gene was instead unexpected. TNF-alfa exerts its best-characterized role in the immune system by inducing a peripheral pro-inflammatory response. Meanwhile, in the CNS, it controls homeostatic brain functions [34], such as neuronal plasticity, excitatory transmission, and neurite growth, but also the proliferation of oligodendrocyte progenitors and remyelination [35]. Thus, further studies could open new perspectives for the comprehension of a possible restorative role of TNF-alfa in the CNS resident cells.

Finally, we evaluated the effects of BAF312 treatment on *NR4A* expression in oligodendrocytes to investigate the potential involvement of these genes in the pro-myelination properties attributed to the drug [7,8,9]. To date, the role of the *NR4A* genes in oligodendrocytes has never been investigated, but it is of great interest also considering that BAF312 specifically binds S1P5, which is predominantly expressed in oligodendrocytes [3]. We have shown for the first time that the *NR4A* genes are expressed in mature oligodendrocytes. However, the in vitro treatment with BAF312 did not modulate their expression, suggesting that BAF312’s pro-myelination properties may be *NR4A*-independent, or that longer treatment may be required to observe modulation. In conclusion, the preliminary findings of our in vitro study suggest that BAF312 may exert its beneficial effects on the CNS by modulating the expression of *NR4A* and TREM-2 in microglia. The revealing of these novel targets induced by BAF312 provides new insights into its cell-specific mechanism of action within the CNS. Despite the use of simplified models, this study lays the foundations for future in-depth studies on human samples.

## 4. Materials and Methods

**Primary culture of human PBMCs**. Buffy coats from HC were obtained from the S.C. Centro Produzione e Validazione Emocomponenti (Città della Salute e della Scienza di Torino, Italy). Specifically, 50 mL of heparinized buffy coat preparation was diluted with an equal volume of phosphate-buffered saline (PBS) at room temperature (RT) and put down slowly over the Lymphoprep (Alere Technologies AS, Oslo, Norway) density gradient to obtain human PBMCs according to the manufacturer’s instructions. PBMCs were counted with Trypan Blue staining, and aliquots containing twenty million PBMCs were stored in a cryopreserving solution of 30% heat-inactivated fetal bovine serum (FBS) (Thermo Fisher Scientific, San Diego, CA, US), 10% dimethyl sulfoxide (DMSO) (Sigma-Aldrich, St Louis, MO, USA), and 60% Roswell Park Memorial Institute (RPMI) 1640 (Thermo Fisher Scientific) at −80 °C until use. On the day of the experiment, after gentle thawing at 37 °C, PBMCs were immediately added to 5 mL of RPMI 1640 (Thermo Fisher Scientific) supplemented with 10% FBS and centrifuged to remove DMSO. The samples were re-suspended in RPMI 1640 medium supplemented with 10% FBS and counted for the experiments. Four million PBMCs were seeded in six-well plates at a concentration of twenty million cells/mL in RPMI 1640 with 10% FBS and incubated at 37 °C with 5% CO_2_. After 2 h, BAF312 (100 nM) was added to the medium. After 24 h, the cells were collected and centrifuged for 10 min at 1500 rpm at 4 °C, the medium was removed, and the dry pellet containing the cells was stored at −80 °C until use. The experiments were performed in triplicate using four buffy coats for each analyzed point.

**N9 microglia cell line**. The murine microglial cell line N9 was originally developed by Prof. P. Ricciardi-Castagnoli [36]. Cells were cultured in Iscove’s Modified Dulbecco’s Medium (IMDM) (Thermo Fisher Scientific) containing 25 mM of HEPES and L-glutamine, supplemented with 5% FBS, 100 IU/mL penicillin, and 100 lg/mL streptomycin (complete medium) in a humidified 5% CO_2_ atmosphere at 37 °C. Eight million cells were cultured in a six-well plate containing three milliliters of complete medium per well. After 2 h, BAF312 (100 nM) and/or lipopolysaccharide (LPS, 1 µg/mL, Sigma-Aldrich) were added to the medium as described in [4]. After 24 h, the cells were collected and centrifuged for 10 min at 1500 rpm at 4 °C, the medium was removed, and the dry pellet containing the cells was stored at −80 °C until use. The experiments were performed in quadruplicate.

**MO3.13 oligodendrocytic cell line**. The MO3.13 cell line (Tebu-bio, Paris, France) is an immortalized human clonal model that expresses the phenotypic characteristics of oligodendrocyte precursor cells. To induce an oligodendrocytic phenotype, we followed the differentiation protocol described by Boscia and colleagues [37]. Briefly, human MO3.13 cells were cultured in an FBS-free chemically defined culture medium composed of Dulbecco’s modified Eagle’s medium (DMEM) supplemented with 500 mg/L insulin, 100 mg/mL human transferrin, 0.52 mg/L sodium selenite, 0.63 mg/mL progesterone, 16.2 mg/mL putrescin, 100 U/mL penicillin, 100 mg/mL streptomycin, 2 mM glutamine, and 100 nM phorbol myristate acetate (PMA) for 7 days in vitro (differentiate medium, PMA T7). Fresh PMA was added every day to the medium. In parallel, human MO3.13 cells were cultured in DMEM supplemented with 10% FBS, 100 U/mL penicillin, 10 mg/mL streptomycin, and 2 mmol/L L-glutamine for 7 days in vitro (FBS T7) as a control. Cells were cultured under a humidified 5% CO_2_ atmosphere at 37 °C. Four million cells were cultured in 175 cm cell culture flasks containing twenty milliliters of differentiation medium until the T7 time point. BAF312 (100 nM) was added to the medium for 24 h at time points T0 and T7. The experiments were performed in quadruplicate.

**Western blot**. Five million cultured MO3.13 cells were lysed for 2 h at 25 °C on an orbital shaker in 500 μL of lysis buffer (50 mM phosphate buffer, 550 mM potassium chloride, 10 mM imidazole, 2.0% maltoside, benzoase nuclease (Sigma-Aldrich), and protease inhibitor cocktail (Sigma–Aldrich); pH 7.4). Crude lysates were centrifuged at 20,000 rpm for 1 h at 4 °C. The supernatant (cleared lysate) was kept for protein quantification and Western blot analysis. Approximately 30 μg protein samples were separated on 4–12% BisTris polyacrylamide gel and transferred onto nitrocellulose membranes. The filters were probed using the following primary antibodies: monoclonal anti-Tubulin (1:1000; Merck Millipore, Burlington, MA, USA), monoclonal anti-CNPase (1:500; Merck Millipore), polyclonal anti-NG2 (1:1000; Merck Millipore), and oligoclonalanti-S100 (1:500; Life Technologies, Carlsbad, CA, USA). The proteins were visualized with peroxidase-conjugated secondary antibodies using the enhanced chemiluminescence system (Thermo Fisher Scientific).

**Immunofluorescence**. MO3.13 cell cultures were fixed in 4% paraformaldehyde phosphate buffer for 30 min. After blocking with PBS 3% bovine serum albumin (BSA) 0.25% triton, the cells were incubated with the monoclonal anti-Tubulin (1:700; Merck Millipore) primary antibody for 24 h. Subsequently, the cells were incubated with Cy3-conjugated anti-mouse IgGs, 1:1000). Images were acquired using a Leica TCS SP5/laser-scanning confocal microscope. Single images were captured with an optical thickness of 0.9 μm and a resolution of 1024 × 1024. All images were obtained with identical laser power settings.

**RNA extraction and gene expression analysis**. Total RNA was extracted with the TRIzol reagent from the PBMC, N9, and MO3.13 cells following the manufacturer’s instructions. Total RNA was reverse-transcribed at a final concentration of 20 ng/μL using a High-Capacity cDNA Reverse Transcription Kit (Thermo Fisher Scientific). Gene expression analysis was performed by real-time PCR using TaqMan^®^ gene expression products (Thermo Fisher Scientific). For primers and probes, Applied Biosystems’ TaqMan^®^ Assay-on-demand-TM gene expression products were used. The expression levels of the target genes were calculated by the normalized comparative cycle threshold (Ct) method (2^−ΔCT^) using glyceraldehyde-3-phosphate dehydrogenase (GAPDH) as a reference gene.

**Statistical analysis**. The normality of distribution was assessed by the Shapiro–Wilk test. The two-tailed t-test, Mann–Whitney U test, one-way ANOVA followed by Tukey’s HSD post hoc test, or Kruskal–Wallis followed by Dunn’s post hoc test were used to compare continuous data between groups, as appropriate. The Benjamini–Hochberg method was used to adjust for multiple comparisons. Statistical significance was considered at *p* values < 0.05. All analyses were carried out using R version 4.1.1. (R Foundation for Statistical Computing, Vienna, Austria).

## Data Availability

Not applicable.
